# Analysis and prediction of serological indicators associated with colorectal interval polyposis after resection

**DOI:** 10.3389/fendo.2025.1634468

**Published:** 2025-07-30

**Authors:** Yuan Lan, Li Zhang, Ren Jun Li

**Affiliations:** Chaohu Hospital of Anhui Medical University, Hefei, China

**Keywords:** colorectal polyps, interval polyposis, resection, prediction of serological indicators, analysis of serological indicators

## Abstract

**Background:**

The examination of the endoscope and the subsequent rediscovery of polyps after endoscopic intervention presents a significant clinical challenge. Multiple factors may contribute to this phenomenon. This study aimed to identify determinants of interval polyposis by analyzing common serological markers and polyp-related variables, ultimately developing a predictive model.

**Methods:**

This retrospective study included 415 patients diagnosed with colorectal polyps who underwent either endoscopic mucosal resection (EMR) or endoscopic submucosal dissection (ESD) at Chaohu Hospital affiliated with Anhui Medical University between December 1, 2022 and December 31, 2023. The primary objective was to evaluate associations between hematologic indicators, polyp-related characteristics, and the risk of rediscovery colorectal polyps. Interval polyposis was defined as the detection of one or more polyps within 18 months after the initial polypectomy, regardless of anatomical location. Follow-up data were obtained through electronic medical records, including demographic information (age, sex, smoking and alcohol use, hypertension, and diabetes history), anthropometric measures (weight, height, BMI), surgical details, polyp features (location, size, number, histological type), and relevant serological parameters.

**Results:**

Significant differences were observed between the non-interval polyposis and interval polyposis groups in terms of hypertension, diabetes, triglycerides (TG) and the number of polyps (P < 0.05). Binary logistic regression analysis identified four independent risk factors: hypertension (OR, 2.741; 95% CI, 1.451-5.179), diabetes (OR, 4.828; 95% CI, 1.943-11.995), number of polyps (OR, 1.211; 95% CI, 1.078-1.361) and triglyceride levels (OR, 1.823; 95% CI, 1.303-2.551).

**Conclusion:**

Hypertension, diabetes, an increased number of polyps and elevated triglyceride levels are independent predictors of colorectal interval polyposis following endoscopic resection. These findings underscore the importance of monitoring blood pressure, blood sugar, triglyceride levels and conducting regular colonoscopic surveillance in high-risk individuals to facilitate early detection and management of interval polyposis.

## Introduction

Colorectal polyps are a common form of intestinal pathology with a steadily increasing incidence worldwide and are widely recognized as precursors to colorectal cancer. According to global statistics, colorectal cancer is the third most prevalent malignancy and imposes a substantial economic burden in terms of treatment and long-term care (1). Endoscopic resection remains the primary therapeutic approach for managing colorectal polyps. However, accurately evaluating the risk of interval polyposis remains a clinical challenge, particularly due to inter-individual variability and uncertainties surrounding the efficacy of postoperative surveillance (2). Therefore, identifying risk factors associated with rediscovery of polyps following colorectal polypectomy holds significant clinical importance.

In recent years, numerous studies have investigated the potential factors contributing to interval polyposis. These include polyp-related characteristics such as size, number, and histological type, as well as patient demographics such as age, sex, and family history (3). Several studies have demonstrated that larger and more numerous polyps are associated with an elevated the risk of rediscovery of polyps, while a positive family history may also increase the likelihood of rediscovery of polyps in certain individuals (4). Despite these findings, comprehensive evaluations of these risk factors and their interrelationships remain limited (2).

This study employs a retrospective cohort design, integrating both statistical methods and machine learning approaches to examine the risk factors influencing interval polyposis after endoscopic resection. By analyzing clinical data through logistic regression and predictive modeling, this research evaluates the impact of various patient- and polyp-related variables on interval polyposis (5). This methodology allows for a nuanced assessment of complex variable interactions and facilitates accurate estimation of interval polyposis risk. The primary objective is to develop a robust predictive model based on common serological markers and polyp characteristics, thereby providing clinicians with valuable tools to guide personalized follow-up strategies and ultimately improve patient outcomes.

## Methods

### Study design

The present investigation employed a retrospective cohort study framework to identify the risk factors associated with rediscovery of polyps following colorectal polypectomy. A total of 799 patients who underwent endoscopic evaluations and were diagnosed with gastrointestinal polyps at the Department of Gastroenterology, Chaohu Hospital affiliated with Anhui Medical University, between December 1, 2022, and December 31, 2023, were screened for further analysis. Following stringent inclusion and exclusion parameters, 415 patients with colorectal polyps were ultimately included in the study, comprising 342 individuals in the non-interval polyposis group (control group) and 73 individuals in the interval polyposis group (experimental group). The data collected encompassed various patient demographics, including age, gender, smoking and alcohol consumption habits, medical history of hypertension and diabetes, as well as anthropometric measurements such as weight, height, and body mass index (BMI). Additional information was gathered regarding the serological laboratory indicators. The serological parameters assessed included fasting blood glucose (FBG), total cholesterol (TC), triglycerides (TG), high-density lipoprotein cholesterol (HDL-C), low-density lipoprotein cholesterol (LDL-C), homocysteine (Hcy), liver enzymes (ALT, AST, and GGT), total and direct bilirubin (TBil, DBil), albumin (ALB), globulin (Glob), albumin/globulin ratio (A/G), renal function markers (BUN, Cr, GFR), uric acid (UA), complete blood counts (WBC, Hb, PLT), coagulation metrics (PT, INR, APTT, FIB, TT, D-D), tumor markers (AFP, CEA, CA199, CA125, CA153, CA724), and ratios such as neutrophil-to-lymphocyte ratio (NLR), platelet-to-lymphocyte ratio (PLR), lymphocyte-to-monocyte ratio (LMR), and systemic immune-inflammation index (SII).

### Inclusion criteria

Eligible participants were required to have a definitive pathological diagnosis of colorectal polyps, undergo at least one follow-up colonoscopy within 18 months post-polypectomy, and provide sufficiently comprehensive clinical and laboratory data.

### Exclusion criteria

Patients were excluded if they had a histopathological diagnosis of high-grade intraepithelial neoplasia or cancer, exhibited severe cardiac or pulmonary dysfunction, or had a history of familial adenomatous polyposis, inflammatory bowel disease, or malignant neoplasms at any anatomical site.

### Colonoscopy procedure

Prior to the examination, patients were instructed to adhere to a liquid diet for 24 hours and abstain from food intake for a minimum of 8 hours. Standardized bowel preparation was achieved using polyethylene glycol. A complete colonoscopy was performed by skilled physicians to ensure adequate bowel cleansing. Visible lesions were excised using endoscopic mucosal resection (EMR) and endoscopic submucosal dissection (ESD) techniques for subsequent biopsy. The specimens were assessed and diagnosed by specialized pathologists using microscopic examination.

### Collection of laboratory hematological indicators and clinical data

Peripheral venous blood samples were obtained within 24 hours following patient admission, while baseline characteristics were extracted from their medical histories. These characteristics encompassed variables such as gender, age, body mass index (BMI), smoking and drinking habits, in addition to the presence of hypertension and diabetes. A positive drinking status was determined if the intake exceeded 30 grams daily. A patient was classified as a smoker if they had engaged in regular smoking for a minimum duration of six months. Hypertension was diagnosed based on blood pressure readings equal to or exceeding 140/90 mmHg, along with the utilization of antihypertensive medications. The diagnosis of diabetes was established when fasting blood glucose levels were equal to or greater than 126 mg/dL, random blood glucose levels were at least 200 mg/dL, hemoglobin A1c (HbA1c) levels reached or surpassed 6.5%, or if the individual had been previously diagnosed and was undergoing treatment with hypoglycemic agents (6).

### Definition of polyp location, number, size, classification, and interval polyposis

Colorectal polyp locations were anatomically categorized into the ascending colon, transverse colon, descending colon, sigmoid colon, and rectum. The count of polyps was primarily determined during colonoscopy. The size of each polyp was defined as the maximum diameter observed during endoscopic examination (in centimeters). The pathology department of the hospital classified the types of polyps in accordance with World Health Organization (WHO) criteria, which included adenomatous polyps, inflammatory polyps, hyperplastic polyps, and hamartomas (7). interval polyposis was defined as the identification of one or more polyps at any point during the follow-up period post-polypectomy, irrespective of their specific anatomical location (8). For the purposes of this study, polyps were categorized into right colon (including the ascending and transverse colon), left colon (comprising the descending colon and sigmoid colon), the overall colon (encompassing both left and right colon), and rectum. In instances where multiple polyps were detected across various locations, the classification and corresponding combinations were based on the right colon, left colon, colon, and rectum. Polyp types were further classified into adenomatous polyps, non-adenomatous polyps (which include inflammatory, hyperplastic, or hamartomatous polyps), and special types (such as lipomas or carcinoids). When multiple polyps of differing types were present, classification and the respective combinations relied on the categories of adenomatous polyps, non-adenomatous polyps, and special types.

### Model development and assessment

In this investigation, the retrospective dataset was randomly divided into training and validation cohorts, allocating 80% of the data for training purposes and the remaining 20% for validation. The training cohort was employed for the development of the predictive model, enabling the model to discern data characteristics and extract pertinent information. Conversely, the validation cohort served to assess the performance of the model. Within the training cohort, both univariate and multivariate logistic regression analyses were performed to identify independent risk factors. Initially, a baseline feature analysis was conducted on each variable to select those with statistically significant outcomes for univariate regression analysis. Variables exhibiting a P-value below 0.1 were incorporated into multivariate analysis, while those with a P-value beneath 0.05 proceeded to further multivariate regression. Following the multivariate analysis, variables with a P-value lower than 0.05 were established as independent risk factors. Additionally, the random forest algorithm was employed to determine the most impactful risk factors, and the data from the validation cohort were utilized to validate the interval polyposis prediction model.

### Statistical evaluation

The statistical evaluation was performed utilizing R software (version 4.2.1). For data that satisfied the criteria for normal distribution and homogeneity of variance, t-tests were applied for inter-group comparisons. In cases where the data followed a normal distribution but did not meet the criteria for variance homogeneity, Welch t-tests were employed. For data that did not conform to a normal distribution, Wilcoxon tests were utilized. Statistically significant variables with P-values less than 0.05 were chosen for logistic univariate analysis. Indicators with P-values under 0.1 were incorporated into multivariate analysis to ascertain independent risk factors, and visual representations were generated using random forest plots and nomograms.

## Results

Baseline characteristics of patients

**Figure 1:**
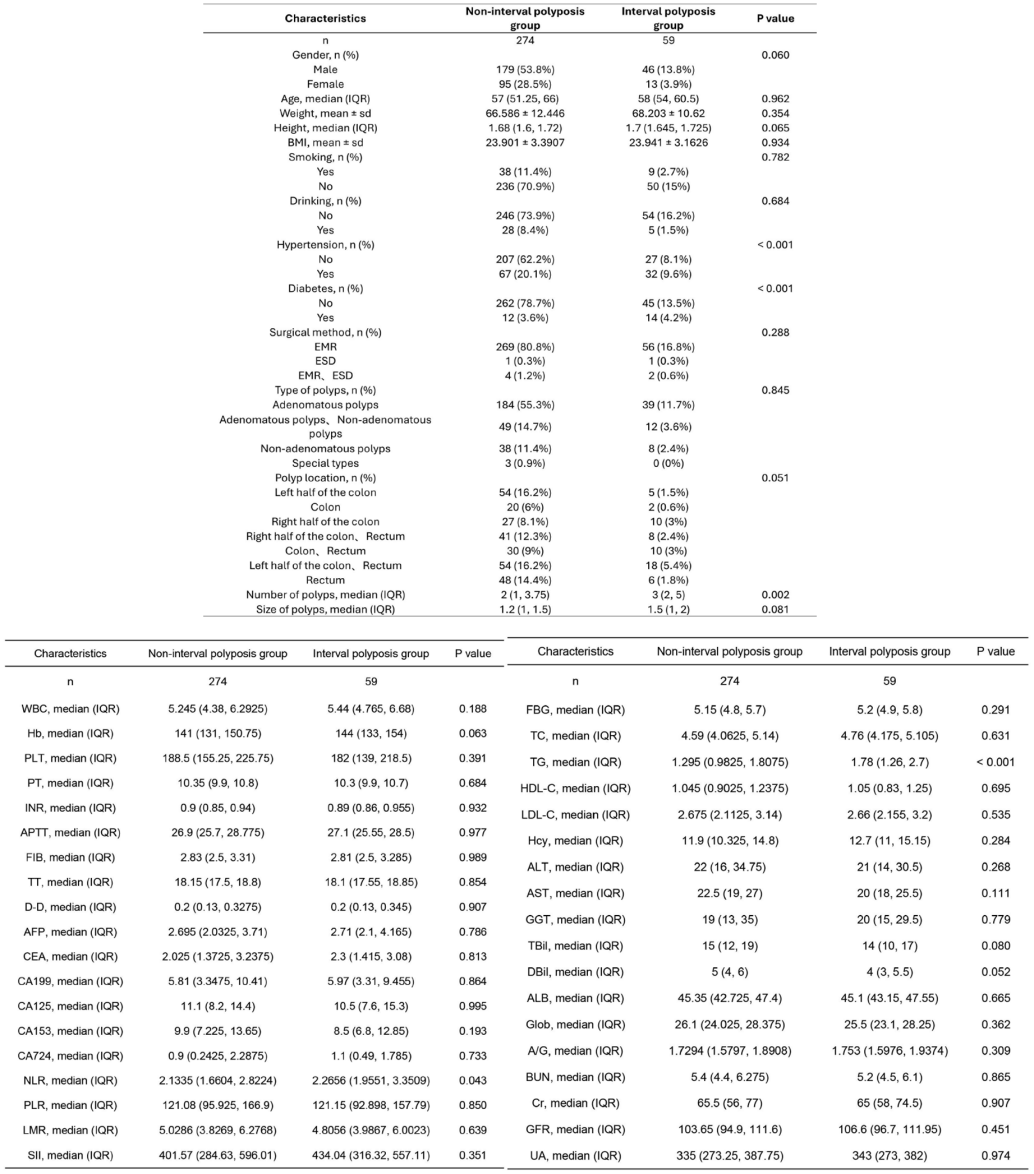
Baseline clinical data of patients in the interval polyposis and non-interval polyposis groups.

Based on the inclusion and exclusion criteria, 59 patients were assigned to the interval polyposis group, and 274 patients were assigned to the non-interval polyposis group in the training cohort. Among all patients in the training cohort, 82.28% were male and 17.72% were female, indicating a higher prevalence of colorectal polyps among male patients. Comparative analysis between the interval polyposis and non-interval polyposis groups revealed statistically significant differences in presence of hypertension, diabetes, triglyceride (TG) levels, polyp count and neutrophil-to-lymphocyte ratio (NLR) (P < 0.05; see **Figure 1**).

### Logistic regression analysis and visualization

**Figure 2:**
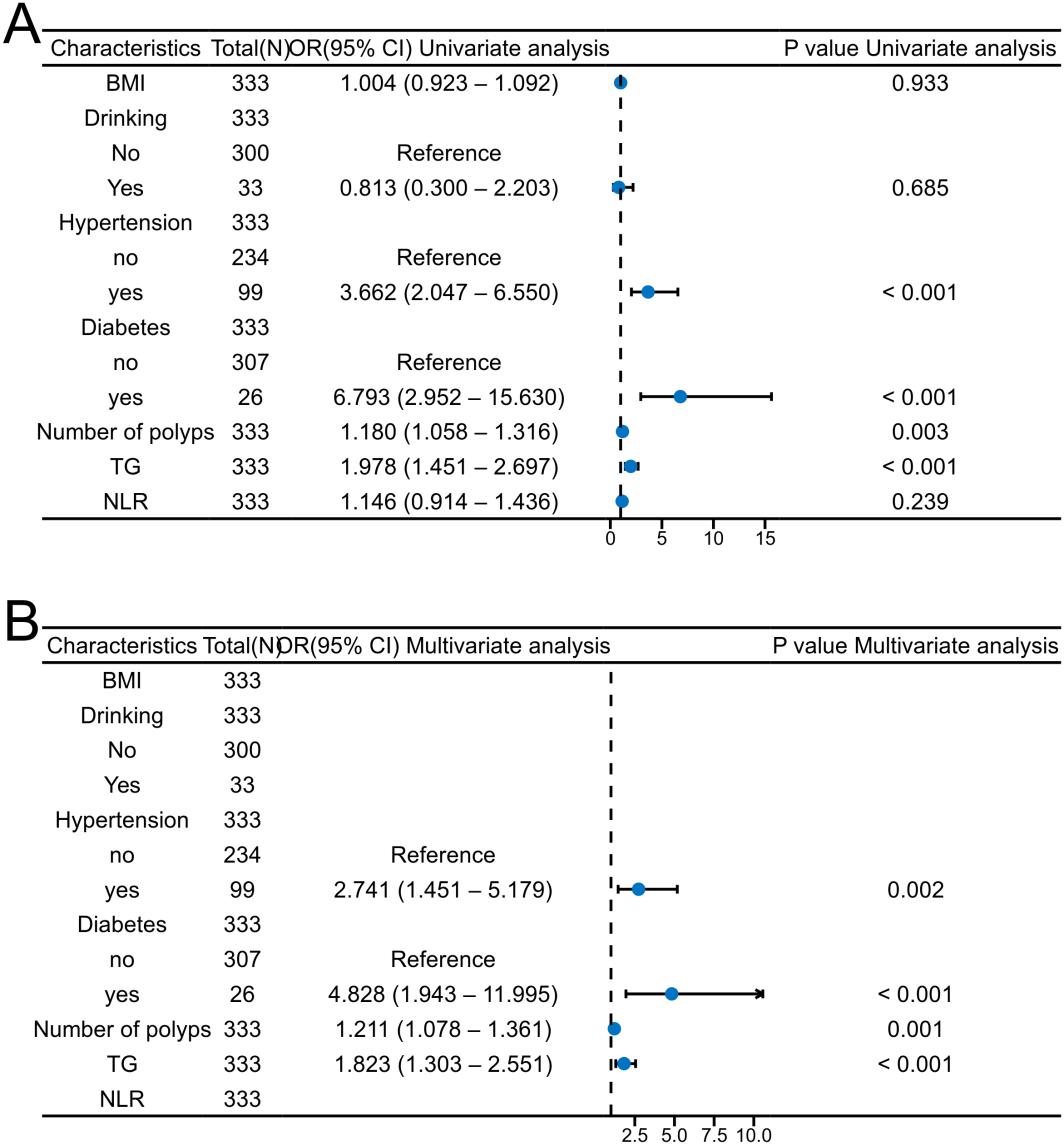
Forest plots depicting logistic regression analyses of interval polyposis. **(A)** univariate, **(B)** multivariate.

Logistic regression was employed to identify factors associated with interval polyposis. In univariate analysis, the following variables were significantly associated with increased rediscovery of polyps risk: hypertension (OR, 3.662; 95% CI, 2.047-6.550), diabetes (OR, 6.793; 95% CI, 2.952-15.630), number of polyps (OR, 1.180; 95% CI, 1.058-1.316), and TG levels (OR, 1.978; 95% CI, 1.451-2.697) (**Figure 2A**).

Variables with P < 0.1 in the univariate analysis were further included in the multivariate logistic regression. The multivariate results demonstrated that hypertension (OR, 2.741; 95% CI, 1.451-5.179), diabetes (OR, 4.828; 95% CI, 1.943-11.995), number of polyps (OR, 1.211; 95% CI, 1.078-1.361) and TG levels (OR, 1.823; 95% CI, 1.303-2.551) were independent risk factors for interval polyposis (**Figure 2B**).

### Random forest model

**Figure 3:**
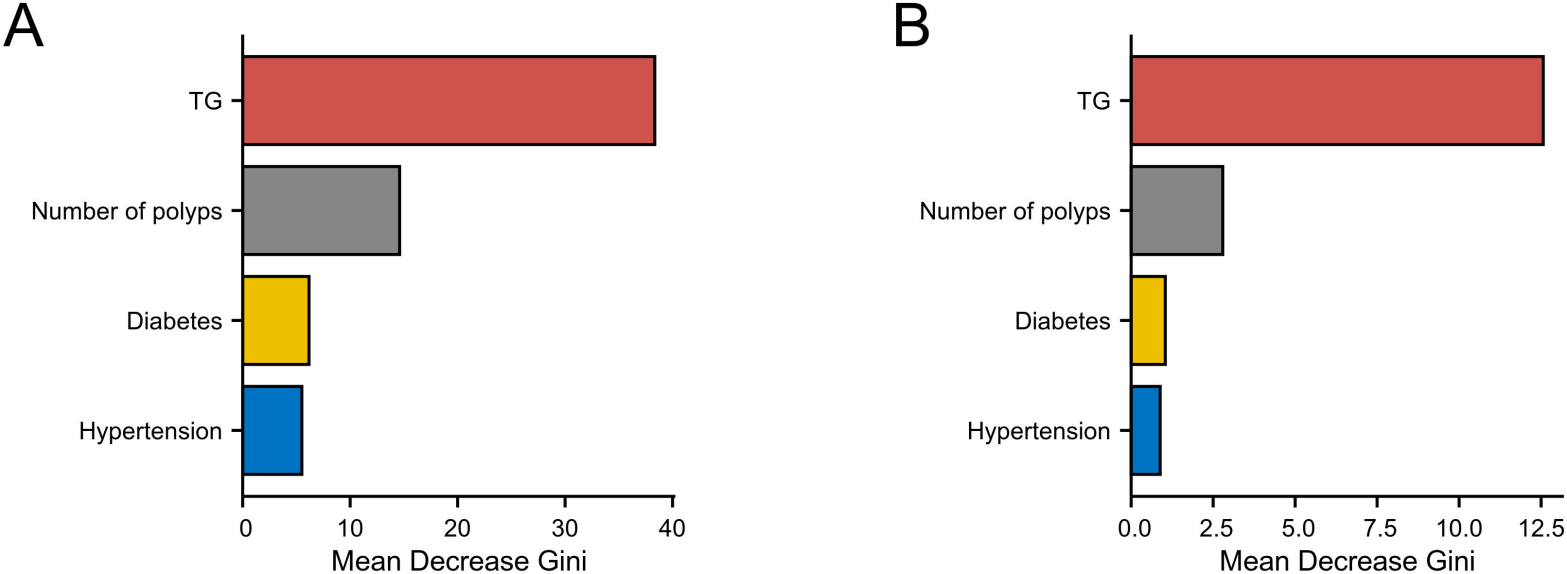
Random forest models predicting interval polyposis. **(A)** training cohort and **(B)** validation cohort.

A random forest algorithm was applied to construct a predictive model for interval polyposis. As illustrated in **Figure 3A**, the model was developed using data from 333 patients in the training cohort, incorporating the variables identified from multivariate logistic regression: hypertension, diabetes, number of polyps, and TG levels. The analysis revealed TG as the most influential predictor of rediscovery of polyps.

The model was subsequently validated using data from 82 patients in the validation cohort. The results (**Figure 3B**) consistently identified TG as the most critical factor influencing rediscovery of polyps, reinforcing the findings from the training set.

### Diagnostic nomogram

**Figure 4:**
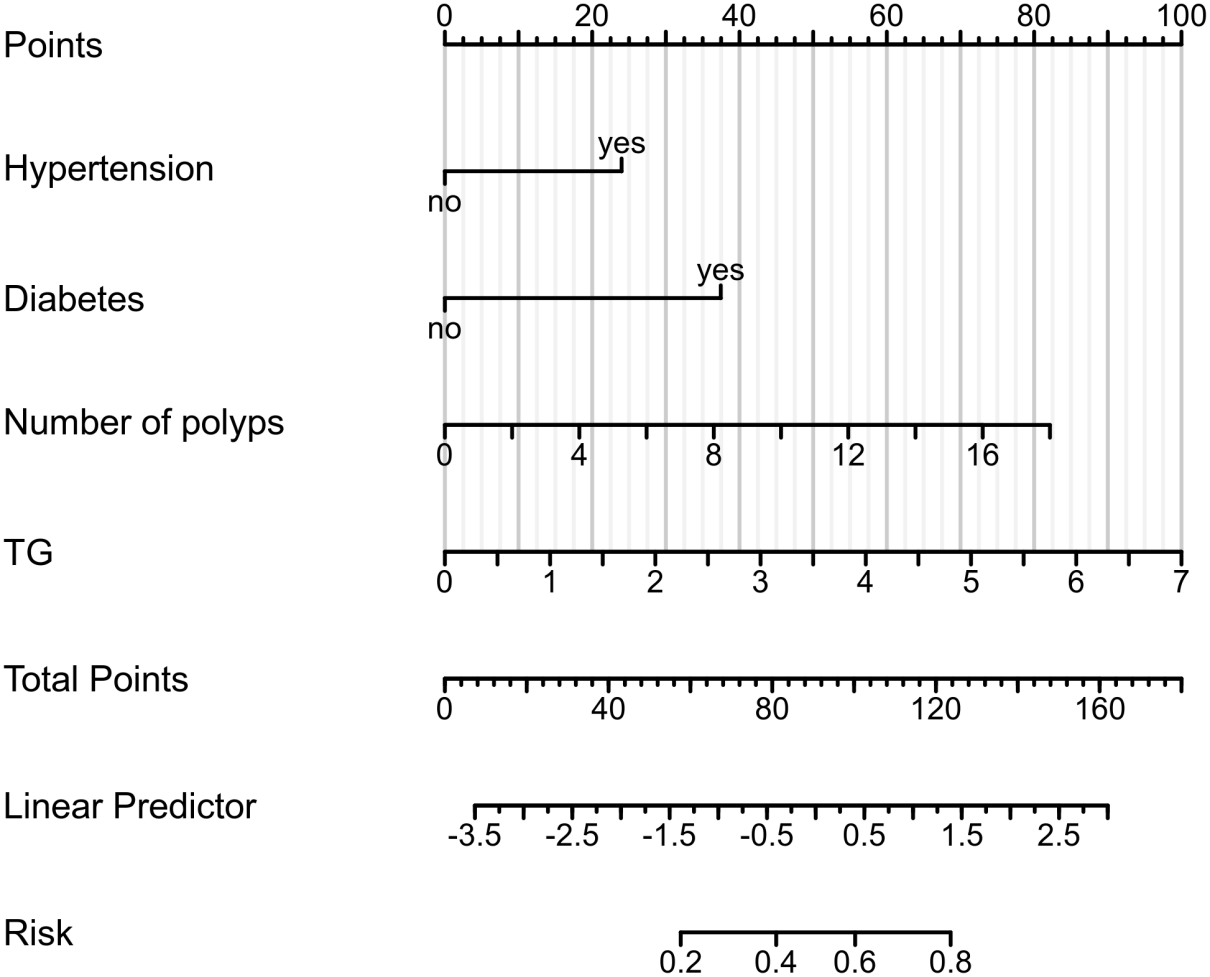
Nomogram predicting colorectal interval polyposis after endoscopic resection.

A diagnostic nomogram was constructed using data from the training cohort (n = 333), integrating four predictors: hypertension, diabetes, polyp count, and TG level. The model’s calibration was evaluated using the Hosmer–Lemeshow goodness-of-fit test, yielding a χ² value of 2.6237 and a P-value of 0.9557 (**Figure 4**). This indicates no significant difference between predicted and observed outcomes, confirming the nomogram’s strong calibration and predictive reliability.

## Discussion

Interval polyposis poses a significant challenge within healthcare, adversely impacting patients’ long-term health and markedly diminishing their quality of life. The global incidence and mortality rates associated with colorectal cancer are alarmingly high, and colorectal polyps are regarded as potential precursors to this malignancy. Existing research indicates that numerous factors—including biochemical markers, lifestyle choices, and genetic predispositions—can influence the likelihood of colorectal interval polyposis. In recent years, advancements in understanding the underlying mechanisms of colorectal interval polyposis have led to increased clinical focus on this matter. Consequently, identifying the risk factors that contribute to interval polyposis and developing effective predictive models are imperative for enhancing clinical practice.

This investigation utilized a retrospective cohort methodology to scrutinize clinical data derived from 415 patients diagnosed with colorectal polyps, with the objective of identifying factors associated with interval polyposis and formulating predictive models. The findings unequivocally indicated that hypertension, diabetes, serum triglyceride concentrations and the quantity of polyps represent independent risk factors for interval polyposis. This research furnishes healthcare professionals with critical insights and establishes a foundation for tailored follow-up and intervention strategies. Consequently, these results contribute to a deeper comprehension of the risk associated with colorectal interval polyposis, ultimately enhancing patient outcomes (1, 2, 4, 9).

The novelty of this study resides in its thorough examination of the risk factors linked to colorectal interval polyposis, particularly the identification of hypertension, diabetes, serum triglyceride levels and the number of polyps as independent predictors. Unlike prior investigations, this study is pioneering in confirming the significance of these biochemical markers in forecasting the likelihood of interval polyposis in humans, aligning with findings from animal studies conducted by Smith et al. (2020), which underscore the distinct mechanistic function of triglycerides (10). Moreover, the results offer clinicians a framework for devising personalized follow-up and management strategies informed by these biochemical markers, an area that has not been adequately addressed in existing literature.

The study’s implications for clinical practice are pronounced. By recognizing patients who are at elevated risk, healthcare providers can institute more rigorous monitoring and intervention strategies to mitigate interval polyposis rates. These findings indicate that serum triglyceride levels and the levels of blood pressure and blood sugar may act as biomarkers for clinical evaluation and monitoring, providing viable treatment avenues for patients with refractory colorectal polyps, thus improving prognostic outcomes and diminishing the risk of cardiovascular complications (4). Furthermore, the results yield significant evidence for policy formulation, advocating for the judicious allocation of medical resources to guarantee that high-risk patients receive enhanced management.

The relationship between adipocytes and tumor cells necessitates further examination. Investigative efforts aimed at mitigating or modifying fat infiltration could pave the way for innovative therapeutic approaches in clinical applications. For instance, cytokines produced by adipocytes may facilitate the progression of intestinal polyps by enhancing the inflammatory response within the tumor microenvironment (11). Consequently, research centered on lipid metabolism is anticipated to become a pivotal area of focus moving forward (12). Such strategies may prove instrumental in decreasing the likelihood of interval polyposis. The specific influences of various lipids on cellular proliferation warrant additional scrutiny, particularly concerning how lipid metabolic pathways may affect cell signaling. Future investigations should center on enhancing patient prognosis through the modulation of lipid metabolism, in conjunction with approaches aimed at reducing blood lipid levels via pharmacological means (13). Furthermore, additional studies are essential to elucidate the relationship between metabolic alterations and the tumor microenvironment, as well as their implications for the colorectal interval polyposis (14).

Despite the considerable ramifications of the findings from this study, certain limitations persist. First and foremost, the relatively small sample size may hinder the generalizability of the outcomes; additionally, the retrospective nature of the study, lacking prospective verification, could introduce selection bias. Furthermore, data collection being confined to a single medical institution restricts the external validity of the results (5). This study details the definition of colorectal interval polyposis, which is clinically operable in terms of time window and site range. However, the current methods still struggle to accurately distinguish between true recurrence and ectopic new polyps, which is the main limitation of this study. Future research should expand the sample size and consider a multi-center design to validate the applicability of the results in different populations, and further explore the relevant influencing factors of true recurrence. At the same time, an in-depth investigation of the potential mechanisms of triglycerides on interval polyposis and other possible influencing factors will provide more comprehensive guidance for the management of colorectal polyps.

In conclusion, this study identifies hypertension, diabetes, triglycerides and the number of polyps as independent risk factors for colorectal interval polyposis, providing crucial guidance for healthcare professionals. These results not only contribute significant scientific knowledge for personalized patient management but also establish a foundation for subsequent related research endeavors. Future studies should delve deeper into the potential mechanisms and interactions of these influencing factors to refine management strategies for colorectal polyps and enhance patient outcomes.

## Conclusion

Hypertension, diabetes, an increased number of polyps and elevated triglyceride levels are independent predictors of colorectal interval polyposis following endoscopic resection. These findings underscore the importance of monitoring blood pressure, blood sugar, triglyceride levels and conducting regular colonoscopic surveillance in high-risk individuals to facilitate early detection and management of recurrent polyps.

## Data Availability

The original contributions presented in the study are included in the article/supplementary material. Further inquiries can be directed to the corresponding author.
